# Structure and Function of Parkin, PINK1, and DJ-1, the Three Musketeers of Neuroprotection

**DOI:** 10.3389/fneur.2013.00038

**Published:** 2013-04-19

**Authors:** Jean-François Trempe, Edward A. Fon

**Affiliations:** ^1^McGill Parkinson Program, Department of Neurology and Neurosurgery, Montreal Neurological Institute, McGill UniversityMontreal, QC, Canada

**Keywords:** Parkinson’s disease, Parkin, PINK1, DJ-1, ubiquitin, phosphorylation, mitochondria, oxidative stress

## Abstract

Autosomal recessive forms of Parkinson’s disease are caused by mutations in three genes: *Parkin*, *PINK1*, and *DJ-1*. These genes encode for proteins with distinct enzymatic activities that may work together to confer neuroprotection. Parkin is an E3 ubiquitin ligase that has been shown to ubiquitinate substrates and to trigger proteasome-dependent degradation or autophagy, two crucial homeostatic processes in neurons. PINK1 is a mitochondrial protein kinase whose activity is required for Parkin-dependent mitophagy, a process that has been linked to neurodegeneration. Finally, DJ-1 is a protein homologous to a broad class of bacterial enzymes that may function as a sensor and modulator of reactive oxygen species, which have been implicated in neurodegenerative diseases. Here, we review the literature on the structure and biochemical functions of these three proteins.

## Introduction

Parkinson’s disease (PD) is a degenerative movement disorder characterized by motor symptoms such as slowness of movement, tremor, rigidity, and postural instability. The motor symptoms are caused by the loss of dopaminergic neurons in the substantia nigra (Schapira and Jenner, 2011; Venderova and Park, 2012). Non-motor symptoms such as loss of olfaction, constipation, and rapid eye movement (REM) sleep disorder are also central to PD and can precede the motor symptoms. Although most cases of PD are idiopathic, there are rare familial forms of the disease that follow Mendelian inheritance patterns that can be traced to single gene mutations (Martin et al., 2011). In particular, autosomal recessive PD is caused primarily by mutations in one of three genes that encode proteins with distinct enzymatic activities: Parkin, PINK1, and DJ-1. Pathogenic mutations in these genes include exonic rearrangements and missense or frameshift mutations (Mata et al., 2004; Tan and Skipper, 2007). Since these mutations lead to a loss of function, i.e., a reduced or abolished activity in the corresponding protein (Martin et al., 2011), we can infer that their normal function(s) prevent cell death. Thus a better understanding of their biochemical activities will help decipher the molecular mechanisms underlying neuronal cell death that causes PD. Here we review recently published studies that significantly advanced our understanding of the structure and biochemical mechanisms employed by Parkin, PINK1, and DJ-1, and highlight the knowledge gaps that need to be filled.

## Parkin

**Figure 1:**
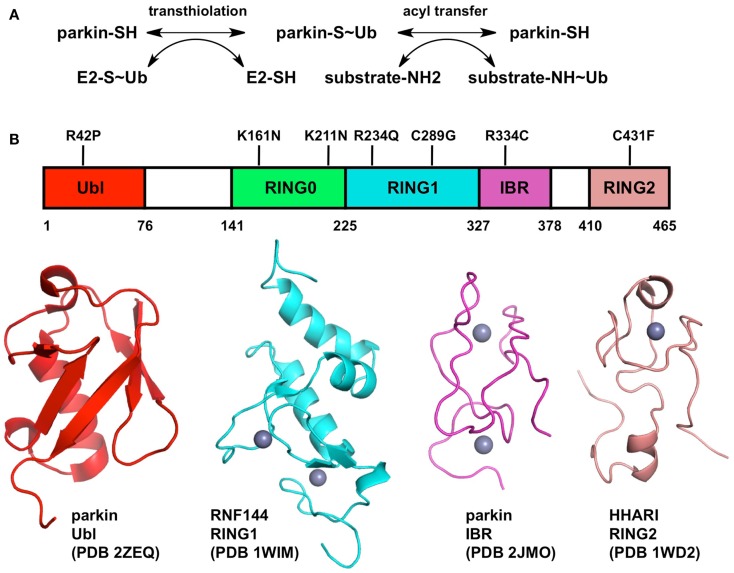
**Biochemical mechanism and structure of Parkin**. **(A)** Two-step mechanism for substrate ubiquitination by Parkin. The first step (transthiolation) is the transfer of ubiquitin from a thioester bond on a cysteine on an E2 enzyme to a thioester bond on Parkin Cys431. The second step (acyl transfer) is the formation of an isopeptide bond on a substrate amino group, typically a lysine side-chain. **(B)** Structure of Parkin. The domain boundaries of full-length human Parkin and selected PD mutations are displayed at the top. The structures of individual domains from Parkin or RBR homologs are shown below. Zinc atoms are displayed as gray spheres.

*Parkin* (*PARK2*) was the first gene associated with autosomal recessive PD (Kitada et al., 1998; Lücking et al., 2000). Parkin is a 52 kDa protein with an N-terminal ubiquitin-like (Ubl) domain followed by a 60 amino acid (a.a.) linker and four zinc-finger domains (Figure 1). Early studies showed that Parkin is an E2-dependent E3 ubiquitin ligase that binds UbcH7 and UbcH8 (Shimura et al., 2000; Zhang et al., 2000). An E3 ligase is an enzyme that catalyzes the transfer of ubiquitin, a small 76 a.a. protein, from an E2 ubiquitin-conjugating enzyme to a protein substrate. Ubiquitinated substrates then undergo different fates depending on the site and type of ubiquitination (described in more details below). However, even today, its cellular function and associated substrates remain controversial. Over the last decade, Parkin has been implicated in numerous seemingly unrelated cellular processes for which many substrates have been suggested (reviewed in Dawson and Dawson, 2010). However, in recent years much attention has converged on Parkin’s role in bioenergetics and mitochondrial quality control pathways (reviewed in Exner et al., 2012). Importantly, Parkin is recruited to depolarized mitochondria where it drives their elimination by autophagy (mitophagy) (Narendra et al., 2008). Interestingly, early studies in Drosophila lacking Parkin revealed its crucial role in mitochondrial homeostasis, and that proteomics studies in Parkin knockout mice showed variations primarily in proteins involved in energy metabolism, mitochondrial function and oxidative stress (Palacino et al., 2004; Periquet et al., 2005). Parkin null mice were recently shown to be acutely resistant to weight gain when fed on a high fat diet due to reduced lipid uptake (Kim et al., 2011). Parkin was also shown to be a p53-target gene that mediates the well-known role of p53 in regulating glucose metabolism (Zhang et al., 2011), and regulates the levels of PGC-1α, an important regulator of mitochondrial biogenesis (Shin et al., 2011). Finally, Parkin was suggested to play a role in the clearance of proteins damaged as a result of dopamine oxidation, as well as in the metabolism of dopamine (Jiang et al., 2004, 2012). These processes all require the ligase activity of Parkin, but the precise molecular mechanisms that underlie these cellular processes remain obscure.

Elucidating the mechanism of ubiquitination by Parkin is crucial for understanding its biological function. The last three zinc-finger domains form an RING1, In-Between, RING2 (RBR) module, which is found in many other E3 ubiquitin ligases such as HHARI, HOIP, HOIL, and Dorfin (Wenzel and Klevit, 2012). A major breakthrough in our understanding of Parkin function was the discovery that RBR proteins use a two-step catalytic mechanism similar to that used by HECT ubiquitin ligases (Wenzel et al., 2011). The discovery stemmed from the observation that the E2 enzyme UbcH7 can only discharge ubiquitin onto cysteine, as opposed to other E2s like UbcH5C that can discharge on cysteine or lysine. Consequently, E3 ligases that are able to use UbcH7 as a conjugating enzyme, like HECT ligases, must bear an acceptor cysteine. In RBR ligases, this cysteine is located in the RING2 domain. Thus in the first reaction (transthiolation), the C-terminus of ubiquitin is transferred from an E2 enzyme’s cysteine to the acceptor cysteine, resulting in the formation of a thioester intermediate (Figure 1). In Parkin, the acceptor cysteine Cys431 is required for its ubiquitin ligase activity (Zhang et al., 2000; Wenzel et al., 2011) and the mutation C431F causes PD (Maruyama et al., 2000), in agreement with its proposed role in catalysis. The second step of the reaction (acyl transfer) involves the transfer of ubiquitin C-terminus from the acceptor cysteine to an amino group on a substrate, forming an isopeptide bond. This HECT-type catalytic model has recently been confirmed for the E3 ligase HOIP, which synthesizes linear polyubiquitin chains (Smit et al., 2012; Stieglitz et al., 2012). Moreover, activation of Parkin upon mitochondrial depolarization induces the HECT-like activity of Parkin, resulting in the formation of an oxyester Parkin∼Ub adduct for the C431S mutant of Parkin (Lazarou et al., 2013). This model has important functional implications for Parkin and may help resolve apparent contradictions in the plethora of biochemical studies on Parkin. For example, binding of E2 enzyme has been reported to require an intact RBR module in cells: deletion of either RING1 or RING2 in Parkin abolished binding (Shimura et al., 2000). Recently, the RING1 domain of HOIP was shown to be required for its strong UbcH7-dependent activity, but not its weaker E2-independent activity, as opposed to RING2 which was required for both functions (Smit et al., 2012). Moreover, the sequence similarity of the RBRs RING1 domain to other E2-binding RING domains, which adopt the cross-brace zinc-binding topology, suggest that it could be the initial docking site of an E2∼Ub complex. Whether this implies that the E2 enzyme binds both RING1 and RING2 simultaneously remains to be investigated.

The structure of an entire RBR module would shine light on our understanding of how E2 enzymes bind Parkin and transfer ubiquitin. Although the structure of full-length Parkin has yet to be determined, structures of individual zinc-finger domains of Parkin and RBR homologs are available (Figure 1). Parkin binds a total of eight zinc atoms and presumably each zinc-finger domain binds two zinc atoms (Hristova et al., 2009). Indeed the structures of the RBR protein RNF144A RING1 (pdb code 1wim, unpublished) and the Parkin IBR (Beasley et al., 2007) have two bound zinc atoms. However, the structure of HHARI RING2 binds only one zinc atom (Capili et al., 2004). To resolve this apparent contradiction, a recent study suggested a novel mode of zinc coordination by Parkin RING2, which comprises two zinc atoms: one bound by Cys418, Cys421, Cys436, Cys441, and the other by Cys446, Cys449, Cys457, and His461 (Rankin et al., 2011). The strong conservation of these eight residues across Parkin orthologs and cysteine mutagenesis studies (Wong et al., 2007) are consistent with this model, which remains to be confirmed by structure determination. The structure of Parkin RING2 could reveal how Cys431 can form a thioester bond with ubiquitin and enable transfer to a substrate amino group.

If the functions of RING1 and RING2 can be inferred from structural and biochemical studies, the functions of the RING0 and IBR domains remain mysterious. The structure of the Parkin IBR reveals a zinc-finger domain with an unusual zinc coordination topology (Beasley et al., 2007). The proximity of its N- and C-termini led Beasley et al. to suggest a role in positioning RING1 and RING2 in proximity, which would be required for the transfer of ubiquitin from a RING1-bound E2∼Ub complex to the acceptor cysteine in RING2. Mutagenesis studies in the zinc-binding cysteines of HOIP IBR domain have indeed shown that the IBR domain is required for its E2-dependent ubiquitin ligase activity (Smit et al., 2012). In this respect, the structure of an entire RBR module would give tremendous insights into the role of the IBR domain. In Parkin, the RBR module is preceded by the RING0 domain, a non-classical zinc-finger that was suggested to adopt a unusual hairpin topology based on weak sequence homology with the cysteine-rich domain of bacterial DnaJ (Rankin et al., 2011). The RING0 domain is unique to Parkin among RBR proteins and is conserved in all its orthologs, suggesting it plays a conserved function specific to Parkin. Future biochemical studies on Parkin should specifically address the role of the IBR and RING0 domain in its ubiquitin ligase activity.

One aspect of Parkin’s function that requires thorough investigation is the type of polyubiquitin chains that it forms. E3 ubiquitin ligases transfer ubiquitin carboxy-terminus from an E2 cysteine thiol onto a protein primary amino group, typically a lysine side-chain ε-amino group or an amino-terminus. The acceptor protein can either be a substrate, or another ubiquitin molecule. The latter give rises to polyubiquitin chains, which can be linked through any of ubiquitin’s seven lysines (K6, K11, K27, K29, K33, K48, or K63-linked chains) or its amino-terminus (linear chains). Using methylated or lysine-free (K0) ubiquitin, two studies on Parkin’s *in vitro* ubiquitin ligase activity claimed that Parkin mediates multiple monoubiquitination, both on itself and on substrates (Hampe et al., 2006; Matsuda et al., 2006). This activity was shown to require only the RING2 domain, as pathogenic mutation in RING1 and IBR, as well as deletions containing only IBR and RING2, did not significantly affect its ubiquitin ligase activity. This contradicted earlier studies that showed both RING1 and RING2 were required for E2-dependent ubiquitination with immunoprecipitated Parkin (Shimura et al., 2000), and that Parkin can make K48 and K63 polyubiquitin chains in cells using one lysine-only ubiquitin variants (Doss-Pepe et al., 2005; Lim et al., 2005). More recently, Durcan et al. (2011) showed that Parkin can form K6, K27, K29, and K63 chains in cells, and that it can form polyubiquitin chains that are deconjugated by ataxin-3 *in vitro* (Durcan et al., 2012). Upon recruitment of Parkin to mitochondria depolarized with the proton ionophore carbonyl cyanide *m*-chlorophenylhydrazone (CCCP), K27, and K63 lysine-only ubiquitin variants were shown to accumulate on mitochondria (Geisler et al., 2010). However, in a separate study, K48 and K63-linked chains were directly detected by mass spectrometry on CCCP-depolarized mitochondria in a Parkin-dependent manner (Chan et al., 2011), and the same chains were also detected on Mfn1 using linkage-specific antibodies (Lazarou et al., 2013). Overall these studies strongly suggest that Parkin has the capacity to synthesize polyubiquitin chains. In the light of the HECT-type model recently proposed for RBR ligases (Wenzel et al., 2011), Parkin could have intrinsic chain type specificity independently of which E2 is used, as was shown for HECT E3 ligases (Kim and Huibregtse, 2009) and the RBR-containing LUBAC complex (Kirisako et al., 2006; Stieglitz et al., 2012). Recently, the IBR-RING2 domains of Parkin were indeed shown *in vitro* to make K48 chains in an E2-independent manner, although the assays were carried out at a high pH (8.8), which can considerably affect the reactivity of the nucleophiles involved in the ubiquitin transfer reactions (Chew et al., 2011). The type of chains and the factors involved in imparting linkage specificity to Parkin thus remain to be determined.

A number of substrates of Parkin have been proposed, but how most of them are recruited to Parkin is unknown. The prime suspect for that function is the Ubl domain, a protein-protein interaction domain that has been shown to bind ubiquitin-interacting motifs (UIM) (Sakata et al., 2003; Fallon et al., 2006), SH3 domains (Trempe et al., 2009) as well as its own C-terminus (Chaugule et al., 2011). A comparison of affinity constants shows that binding to the SH3 domain of endophilin-A and C-terminal of Parkin is in the 1–10 μM range (Trempe et al., 2009; Chaugule et al., 2011), whereas binding to UIMs is >100 μM (Safadi and Shaw, 2010). Structurally, the Ubl interacts with all its ligands via the same surface centered on Ile44. A number of PD mutations are found in the Ubl domain, implying that it is essential to the function of Parkin, although some mutations such as R42P unfold the domain, which can lead to its aggregation and degradation (Henn et al., 2005; Safadi et al., 2011). The Ubl domain is required for some proposed functions of Parkin, such as endophilin-A ubiquitination (Trempe et al., 2009) and the regulation of lipid uptake through ubiquitin-mediated stabilization of the CD36 lipid transporter (Kim et al., 2011). Although it is not known whether the Ubl binds a mitochondrial ligand, it has been shown to be required for efficient mitochondrial recruitment and mitophagy in two reports (Narendra et al., 2010; Shiba-Fukushima et al., 2012), but not in others (Geisler et al., 2010; Matsuda et al., 2010). This contradiction can be resolved by considering that Parkin without the Ubl domain has slower recruitment kinetics, as shown recently by Shiba-Fukushima et al. (2012). But since Parkin can be recruited to the mitochondria without the Ubl, it seems unlikely that recruitment to mitochondrial substrates such as mitofusin and Miro is mediated by the Ubl domain. However, PD mutants in the RING0 domain (K161N, K211N, C212Y) have strongly impaired mitochondrial recruitment and clearance activity (Geisler et al., 2010; Matsuda et al., 2010; Narendra et al., 2010), raising the possibility that the RING0 domain could mediate substrate recruitment on mitochondria.

The activity of Parkin appears to be regulated at multiple levels. The first is that Parkin appears to be auto-inhibited in its basal state. Deletion or mutations in the Ubl domain, as well as addition of Ubl-binding ligands or N-terminal tags, increase substantially the autoubiquitination activity of Parkin (Chaugule et al., 2011). Chaugule et al. proposed that Parkin auto-inhibition is maintained by the interaction of the Ubl with the C-terminal domains of Parkin, although exactly how this is achieved is unclear. ΔUbl Parkin does not bind E2 enzymes more strongly, but it exhibits slightly faster E2∼Ub discharging kinetics (Chaugule et al., 2011). Identifying the Ubl-binding site on Parkin’s C-terminal domains will help resolve the mechanism of auto-inhibition.

The second level of Parkin regulation is through post-translational modifications. There is strong evidence that phosphorylation plays a key role in the regulation of Parkin. First, PINK1 kinase activity is required for the recruitment of Parkin to depolarized mitochondria and for the activation of its ubiquitin ligase activity (Geisler et al., 2010; Matsuda et al., 2010; Narendra et al., 2010; Vives-Bauza et al., 2010; Lazarou et al., 2013). FLIM studies suggest that Parkin and PINK1 are in close proximity on depolarized mitochondria (Vives-Bauza et al., 2010). Immunoprecipitations experiments have shown that Parkin and PINK1 are part of the same complex (Xiong et al., 2009; Sha et al., 2010; Vives-Bauza et al., 2010), but recent blue native PAGE (Lazarou et al., 2012) and size-exclusion chromatography (Thomas et al., 2011) studies showed that PINK1 and Parkin do not form a complex. Thus, whether PINK1 and Parkin bind each other remains controversial. However, *in vitro* experiments have demonstrated that PINK1 phosphorylates Ser65 in the Ubl domain, which increases its ubiquitin ligase activity (Kondapalli et al., 2012). Mutation of Ser65 to Ala significantly impaired, but did not inhibit, translocation of Parkin to depolarized mitochondria, suggesting that Ser65 phosphorylation primes Parkin for recruitment (Shiba-Fukushima et al., 2012). This effect could be mediated by the disruption of the autoinhibitory interaction of the Ubl domain (Chaugule et al., 2011). Phosphorylation can also negatively regulate Parkin: two groups found that Tyr143 phosphorylation by the protein tyrosine kinase c-Abl reduces Parkin ubiquitination activity *in vitro* and in cells (Ko et al., 2010; Imam et al., 2011). Parkin inactivation can also be brought upon oxidative stress (Winklhofer et al., 2003) and covalent modifications of its essential cysteines, either through S-nitrosylation or dopamine quinone adducts formation (Chung et al., 2004; LaVoie et al., 2005). Finally, Parkin levels may be regulated by autoubiquitination, and indeed its coupling to the deubiquitinating enzyme ataxin-3 appears to regulate its stability in cells (Durcan et al., 2011, 2012). Moreover, activation of Parkin upon mitochondrial membrane depolarization induces its degradation through the proteasome, suggesting that the auto-inhibition of Parkin may protect itself from ubiquitin-mediated degradation (Rakovic et al., 2012).

## PINK1

**Figure 2:**
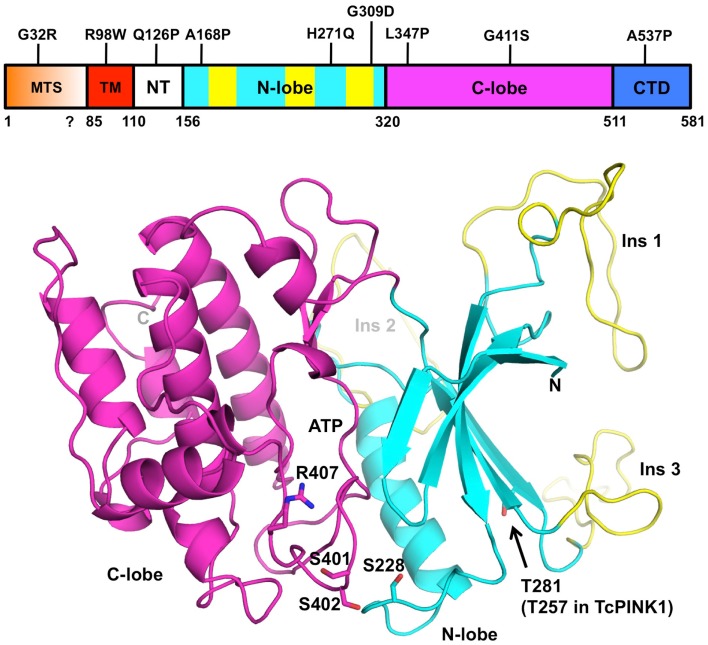
**Structural model of PINK1**. The domain boundaries of full-length human PINK1 and PD mutations are displayed at the top. The domains are colored as follows: mitochondrial targeting sequence (MTS, orange), transmembrane helix (TM, red), N-terminal regulatory region (NT, white), N- and C-terminal lobes (cyan and magenta), C-terminal domain (CTD, blue). The three PINK1-specific insertions are colored in yellow. The coordinates for human PINK1 156–511 (bottom cartoon) were obtained from the Protein Model DataBase (PM0077187). The N- and C-terminal lobes are colored in cyan and magenta, respectively. The ATP binding site is located in the cleft between the two lobes. The three phosphorylation sites (Ser228, Ser402, Thr281) and activation loop residues (Ser401, Ser402, and Arg407) are shown as sticks. The model does not comprise the N-terminal (112–155) and C-terminal (512–581) regions of the soluble domain, which cannot be modeled by homology.

The *PINK1* (*PARK6*) gene was first described as PTEN-induced putative kinase 1, a ubiquitous gene product whose expression was abolished in ovarian tumor tissues due to defect in PTEN signaling (Unoki and Nakamura, 2001). Later, mutations in *PINK1* were found to cause autosomal recessive PD (Valente et al., 2004). Mammalian PINK1 is a 581-residue protein, with an N-terminal mitochondrial targeting sequence, a transmembrane helix, a serine/threonine kinase domain, and a C-terminal domain of unknown function (Figure 2) (Beilina et al., 2005). Studies in Drosophila showed that PINK1 has a role in the maintenance of mitochondria, and this role is intimately linked to Parkin (Clark et al., 2006; Park et al., 2006). PINK1 was shown to regulate HtrA2, a mitochondria protease which plays a role in mitochondrial homeostasis (Plun-Favreau et al., 2007). PINK1 also regulates mitochondrial morphology in mammalian cells (Poole et al., 2008), and is essential for the recruitment of Parkin to mitochondria in cultured immortalized cells (Geisler et al., 2010; Matsuda et al., 2010; Narendra et al., 2010; Vives-Bauza et al., 2010; Lazarou et al., 2013) as well as in neurons (Wang et al., 2011; Joselin et al., 2012). Physiologically, PINK1 deficiency leads to an altered mitochondrial calcium buffering capacity and impaired respiration caused by a reduced provision of electron transport chain substrates (Gandhi et al., 2009). Recently, vitamin K2 was found to rescue PINK1 deficiency through its capacity to carry electrons (Vos et al., 2012). Thus PINK1 appears to have a clear role in mitochondrial maintenance, which contributes to neuronal survival.

Many of the mitochondrial functions carried by PINK1 depend on its kinase activity, which has been the focus of a number of studies. For example, whereas wild-type PINK1 can rescue Parkin mitochondrial localization in *PINK1*^−/−^ mouse embryonic fibroblasts (MEFs), a kinase-dead mutant of PINK1 cannot (Geisler et al., 2010; Matsuda et al., 2010; Narendra et al., 2010; Vives-Bauza et al., 2010). Therefore a better understanding of PINK1 kinase activity will give much insight into the function of PINK1. *In vitro*, recombinant PINK1 from different species can phosphorylate itself as well as artificial substrates such as α-casein or the myelin basic protein (Beilina et al., 2005; Silvestri et al., 2005; Woodroof et al., 2011). However, the degree of activity appears to be dependent on the PINK1 construction used, the expression system and the species. In one instance, recombinant human PINK1 (112–496), which was expressed in *Escherichia coli* and lacked the C-terminal domain, was found to have increased activity compared to a construct that contained the entire C-terminus (112–581) (Silvestri et al., 2005). The human PINK1 kinase domain (112–496) was also shown to be active in two earlier reports (Nakajima et al., 2003; Beilina et al., 2005). However, later studies obtained opposite results, although the construct boundaries were different: human PINK1 (148–581) expressed in Sf9 insect cells was more active than the isolated kinase domain (148–515) (Sim et al., 2006). In a survey of kinase activity across different PINK1 orthologs, *Tribolium castaneum* (Tc) PINK1 was the most active and human PINK1 was completely inactive (Woodroof et al., 2011). Moreover, TcPINK1 128–570 was more active than 155–570, and 155–486 had no activity. Overall, these studies suggest that the segments located at the N- and C-termini of the kinase domain have regulatory functions, and that the human PINK1 may be auto-inhibited. The importance of these regions in regulating PINK1 is supported by the clustering of several PD mutations within these regions (Sim et al., 2012).

Although there is no crystal structure of PINK1 available, the similarity of its kinase domain to other serine/threonine kinases led a number of groups to perform homology modeling (Beilina et al., 2005; Mills et al., 2008; Cardona et al., 2011; Sim et al., 2012). The protein is most similar to the calmodulin-dependent kinase family, with which it shares a number of structural features (Figure 2). Overall, the kinase domain consists of N- and C-terminals lobes, which can be further subdivided into smaller subdomains found in most kinases. The cleft between the two lobes harbors the catalytic and ATP:Mg^2+^ binding sites (Cardona et al., 2011). Three loop insertions in the N-terminal lobe are unique to PINK1 and contain PD mutations, but the function of these inserts is unknown. The PINK1 kinase domain also contains an activation loop with two serine residues (Ser401-Ser402) whose phosphorylation was shown to be activating in other kinases (Nolen et al., 2004). The activation loop also contains Arg407, a PD mutation site and a potential site of interaction with a phosphorylated serine.

Similarly to Parkin, PINK1 is activated in cells upon mitochondrial membrane depolarization, but the mechanism of activation remains unknown. Activation appears to be linked to its abundance, localization and processing. Indeed, endogenous PINK1 is found at low levels in cells as a result of its high turnover rate (Lin and Kang, 2008; Narendra et al., 2010). PINK1 is normally imported through the mitochondrial outer and inner membranes, where it is successively cleaved by the mitochondrial processing peptidase (MPP) and PARL/AFG3L2 (Jin et al., 2010; Deas et al., 2011; Meissner et al., 2011; Greene et al., 2012). The MPP cleavage site in PINK1 is unknown but is probably located in the residue range amino acids 20–70, and PARL cleaves PINK1 between Ala103-Phe104 in the transmembrane helix (Jin et al., 2010; Deas et al., 2011; Kondapalli et al., 2012). This hydrophobic transmembrane helix was also suggested to act as a stop signal that prevents further translocation into the matrix of the mitochondria (Zhou et al., 2008; Lin and Kang, 2010; Becker et al., 2012). The resulting 52 kDa processing fragment may then undergo multiple fates: in its soluble form it is exported to the cytosol where it is degraded by the proteasome (Lin and Kang, 2008; Narendra et al., 2010; Greene et al., 2012). However, a recent study showed using an *in vitro* import assay that the 52 kDa form can also be retained at the outer mitochondrial membrane (OMM) via its C-terminal fragment (Becker et al., 2012). Because mitochondrial import of PINK1 is driven by the inner membrane proton gradient, membrane depolarization leads to the accumulation of unprocessed PINK1 at the OMM (Narendra et al., 2010; Becker et al., 2012; Greene et al., 2012). Prior studies had shown that the kinase domain of PINK1 faces the cytoplasm in its unprocessed mitochondria-tethered form, which would enable PINK1 to phosphorylate both cytosolic and OMM proteins (Zhou et al., 2008; Lin and Kang, 2010). The complex membrane potential-dependence of PINK1 processing and localization would thus serve to link PINK1’s activity to the state of a mitochondrion, thereby suggesting a mechanism for the detection of damaged mitochondria.

Recent studies point to the role of oligomerization and autophosphorylation in the activation of PINK1. Indeed, upon accumulation on the OMM, PINK1 forms a large 700 kDa multimeric complex with the translocase of the outer membrane (TOM) complex (Becker et al., 2012; Lazarou et al., 2012). This was proposed to allow for the rapid reimport and processing of PINK1 upon reestablishment of the membrane potential. Moreover, PINK1 autophosphorylates when it accumulates on mitochondria (Okatsu et al., 2012). In this study, Okatsu et al. showed that Ser402 in the activation loop was required for autophosphorylation, although the authors could not detect direct phosphorylation of this residue, but detected Ser228 phosphorylation. The double mutant S228A/S402A failed to rescue GFP-Parkin mitochondrial translocation in *PINK1*^−/−^ MEFs, but S228D/S402D did rescue, suggesting that PINK1 autophosphorylation is required for Parkin activation. In a separate study, Kondapalli et al. (2012) found that Thr257 in TcPINK1 (Thr282 in human) is phosphorylated upon mitochondrial membrane depolarization, but mutation of that residue did not affect Parkin activation. The ability of PINK1 to multimerize and autophosphorylate is reminiscent of the activation mode of the homologous calmodulin-dependent kinases, which form dodecameric ring structures where the monomers phosphorylate each other upon binding Ca^2+^-bound calmodulin (Rellos et al., 2010; Chao et al., 2011). Interestingly, Sha et al. (2010) observed that PINK1, affinity-purified from mammalian cells, could only phosphorylate Parkin in the presence of Ca^2+^. Considering the role of PINK1 in regulating Ca^2+^ buffering by mitochondria (Gandhi et al., 2009), future studies should address how Ca^2+^ influences the activation of PINK1.

The identity of PINK1 substrate(s) remains a subject of much debate. Studies using artificial peptide substrates indicated that proline was the preferred amino acid at the +1 position (Woodroof et al., 2011), and PREDIKIN predicts a weak consensus phosphorylation site (Sim et al., 2012). Early studies identified the mitochondrial chaperone TRAP1 as a ligand and phosphorylation substrate of PINK1 (Pridgeon et al., 2007). More recently, Miro, a protein involved in the axonal transport of mitochondria, was also found to be phosphorylated by PINK1, resulting in Parkin-mediated ubiquitination and degradation of Miro (Wang et al., 2011). Although an earlier study failed to detect Parkin phosphorylation by PINK1 *in vitro* (Vives-Bauza et al., 2010), Parkin was later reported to be phosphorylated by PINK1 *in vitro* and *in vivo* (Sha et al., 2010). More recently, *in vitro* assays with recombinant proteins failed to detect significant phosphorylation of either TRAP1 or Miro, but detected significant phosphorylation of Parkin on Ser65 in the Ubl (Kondapalli et al., 2012). Parkin Ser65 phosphorylation was later confirmed and showed to be required for efficient Parkin mitochondrial translocation (Shiba-Fukushima et al., 2012). Therefore it appears that Parkin could be the main substrate of PINK1, but the structural basis for PINK1 kinase activity targeting the Parkin Ubl remains unknown.

## DJ-1

The *DJ-1* (*PARK7*) gene was first identified as an oncogene (Nagakubo et al., 1997), and similarly to PINK1, was later associated with familial PD (Bonifati et al., 2003). The protein has neuroprotective activity and affects sensitivity to oxidative stress (Canet-Aviles et al., 2004; Martinat et al., 2004). The effect could be mediated through localization to mitochondria, where it can reduce the oxidative stress induced by inhibitors of the respiratory chain such as rotenone (Canet-Aviles et al., 2004; Blackinton et al., 2009). DJ-1 deficiency leads to altered mitochondrial morphology and increases the production of reactive oxygen species (ROS) as a results of altered mitochondrial dynamics (Irrcher et al., 2010). DJ-1-dependent mitochondrial defects can be rescued by addition of a cell-permeable glutathione precursor or Parkin/PINK1 overexpression (Thomas et al., 2011). Recently, DJ-1 was found to negatively regulate PINK1-dependent Parkin translocation to depolarized mitochondria in neurons, as a result of its ability to control ROS generation (Joselin et al., 2012). Overall, functional data indicate that DJ-1 protects cells from oxidative stress caused by ROS, but how this is achieved at the molecular level is unclear.

**Figure 3:**
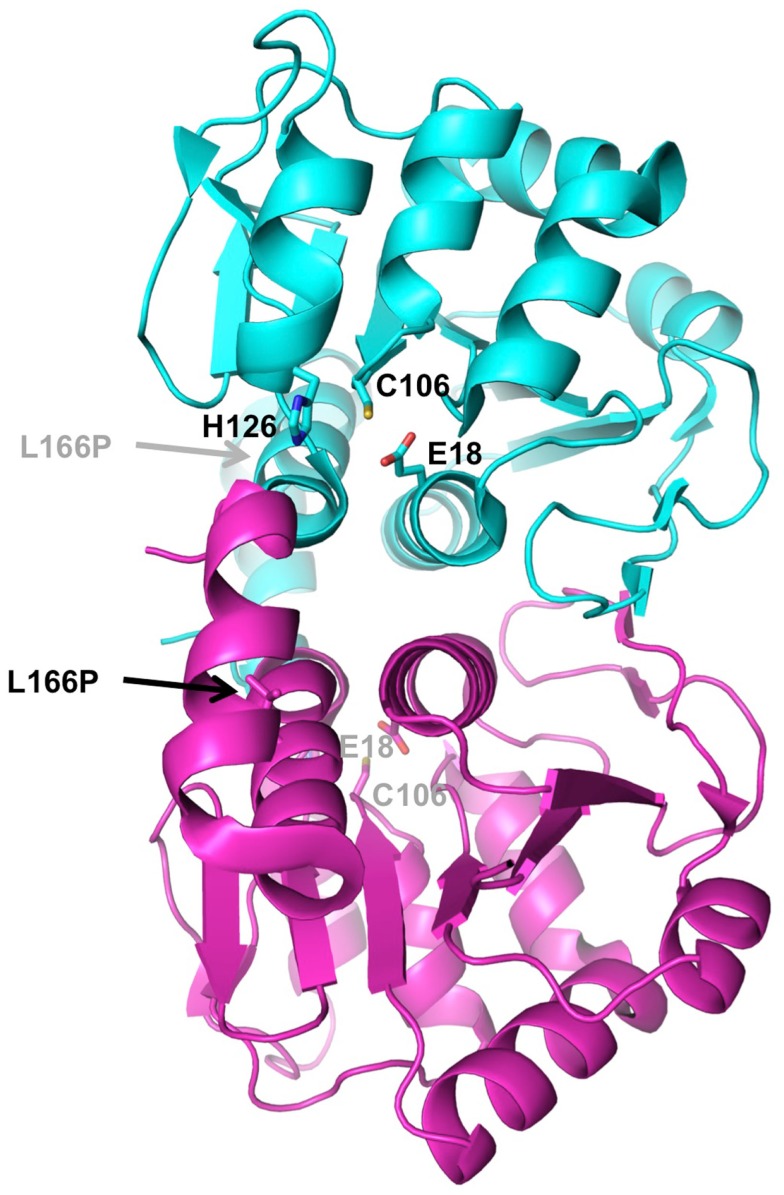
**Structure of DJ-1**. The crystal structure of human DJ-1 (PDB 1P5F) was used to generate this dimer, which represents the biologically active unit. The two subunits are colored in cyan and magenta. Residues of the active site catalytic triad (Glu18, Cys106, His126) are shown as sticks. The location of the PD mutation that disrupts dimerization (L166P) is indicated by arrow.

The DJ-1 protein forms a single 20 kDa domain homologous to a number of bacterial enzyme families such as ThiJ, a protein involved in thiamine biosynthesis, and the Pfp1 protease (Bonifati et al., 2003), as well as the YajL redox-sensitive chaperone (Gautier et al., 2012). Its three-dimensional structure has been determined by several groups nearly 10 years ago (Honbou et al., 2003; Lee et al., 2003; Tao and Tong, 2003; Wilson et al., 2003), and shows a compact globular domain with an active site Cys106 particularly sensitive to oxidation (Figure 3). In solution, DJ-1 forms a stable dimer (Wilson et al., 2003). The PD mutation L166P, which abolishes dimerization, also abrogates its neuroprotective activity, suggesting that dimerization is essential to its function (Olzmann et al., 2004).

In spite of this wealth of structural and homology data, the precise biochemical function of DJ-1 has surprisingly not yet been ascribed. Its closest homolog in bacteria, ThiJ, catalyses the phosphorylation of hydroxymethylpyrimidine (HMP) to HMP-phosphate, a thiamine derivative (Mizote et al., 1996). Under oxidative conditions the mammalian DJ-1 forms a cysteine-sulfinic acid adduct *in vitro* and in cells, which has been proposed to drive mitochondrial localization and neuroprotection (Canet-Aviles et al., 2004; Blackinton et al., 2009). The molecular consequences of adduct formation remain to be investigated, but it likely modifies one of its many proposed enzymatic activities. DJ-1 bears homology to bacterial proteases and although its Cys/His/Glu catalytic triad is not optimally positioned for catalysis (Wilson et al., 2003), DJ-1 was nonetheless shown to have proteolytic activity upon removal of its inhibitory C-terminal helix (Chen et al., 2010). DJ-1 also prevents thermal aggregation of citrate synthase *in vitro* as well as α-synuclein fibril formation *in vitro* and *in vivo* (Shendelman et al., 2004), suggesting that DJ-1 could have chaperone activity similarly to the YajL bacterial chaperone (Gautier et al., 2012). Interestingly, this chaperone activity is mediated only by DJ-1 with an oxidized Cys106, thus providing a potential molecular mechanism for the detection of ROS. An interesting new lead for DJ-1 comes from the recent discovery of its glyoxalase activity (Lee et al., 2012). Glyoxals are small α-oxoaldehyde molecules produced notably as a result of glucose oxidation in conditions of rapid glycolysis. Glyoxals are toxic to the cell because they react with proteins to form advanced glycation end-products (AGEs), which have been implicated in a number of neurodegenerative diseases including PD (Castellani et al., 1996). DJ-1 was shown to protect MEFs and SH-SY5Y cells, as well as *C. elegans*, against glyoxals treatment. However, cells being already equipped with two glutathione-dependent glyoxalases that remove reactive glyoxals, it is unclear what additional roles could be played by DJ-1.

In addition to its proposed enzymatic activities, DJ-1 was also found to bind many biological macromolecules, including RNA (van der Brug et al., 2008), Cezanne (McNally et al., 2011), and Bcl-XL (Ren et al., 2011). All of these interactions were shown to depend on the oxidation state and/or the presence of Cys106. However, considering the high reactivity of the active site cysteine, it is possible that these interactions are mediated through non-specific covalent bond formation between the ligands and Cys106 in DJ-1.

At present, it is difficult to assign a specific biochemical activity to its function *in vivo* because all enzymatic activities described for DJ-1 rely on the active site Cys106 reactivity. Indeed, Cys106 has a depressed pKa as a result of the proximity of Glu18, which makes it a strong nucleophile (Witt et al., 2008). However, the different activities proposed for DJ-1 are not necessarily exclusive: both protease and glyoxalase activities would be diminished upon oxidation of Cys106 or formation of a sulfinic acid derivative. An altered enzymatic activity even provides a molecular mechanism for the proposed role of DJ-1 as a cellular redox sensor. Finally, dopamine-derived quinones were recently shown to covalently modify Cys106, which could potentially impair any of its biological activities (Girotto et al., 2012). This is especially relevant to PD, as oxidized dopamine side-products have been suggested to play a role in the degeneration of dopaminergic neurons (Hastings et al., 1996). Future work should therefore aim to identify the biochemical function of DJ-1 most relevant to its neuroprotective role.

## Concluding Remarks

Over the past 15 years, deciphering the biochemical basis for the function of Parkin, PINK1, and DJ-1 has led to great advances in our understanding of the pathways and mechanisms involved in PD. One challenge for the future will be to integrate the functions of these three enzymes into a single coherent model of neuroprotection. In recent years, a picture has emerged wherein the hub of all three enzymes appears to be mitochondrial quality control through regulation of fusion/fission and ROS generation. Indeed, deficiency in any of these three genes has been shown to affect mitochondrial morphology and dynamics (Deng et al., 2008; Poole et al., 2008; Irrcher et al., 2010), and these defects can be rescued by overexpression of any of the other three genes (Thomas et al., 2011). Mitochondrial fragmentation caused by a-synuclein overexpression can also be rescued by overexpression of wild-type Parkin, PINK1, or DJ-1, but not their functionally deficient mutants (Kamp et al., 2010). Overexpression of either PINK1 or Parkin lead to mitochondrial arrest in the axons of cultured neurons, an effect which depends on the kinase activitiy of PINK1 and the presence of Parkin in the case of PINK1 overexpression (Wang et al., 2011). Finally, the recruitment of Parkin to depolarized mitochondria in neurons is influenced by oxidative stress levels and the presence of wild-type, but not C106A, DJ-1 (Joselin et al., 2012). These cross-talks between Parkin, PINK1, and DJ-1 activities are most likely mediated through OMM proteins involved in fusion/fission such as Mfn1/2 or Fis1 (Yang et al., 2008; Cui et al., 2010; Rakovic et al., 2011; Zhang et al., 2012), or regulators of motility such as Miro1/2 (Wang et al., 2011). Future studies should examine in more details the functional relationships between these three proteins at the biochemical level.

Another important goal is to gain more detailed structural insight for Parkin and PINK1, which will lead to a better understanding of their biochemical functions and regulatory mechanisms. Taken together, the structure and function of this trio of enzymes will lead to the design of new therapies that will enhance and/or correct the function of these proteins, when they are defective in PD.

## Conflict of Interest Statement

The authors declare that the research was conducted in the absence of any commercial or financial relationships that could be construed as a potential conflict of interest.
